# Effect of a novel synbiotic on *Streptococcus mutans*

**DOI:** 10.1038/s41598-020-64956-8

**Published:** 2020-05-14

**Authors:** Mohammed Nadeem Bijle, Prasanna Neelakantan, Manikandan Ekambaram, Edward C. M. Lo, Cynthia Kar Yung Yiu

**Affiliations:** 1 Paediatric Dentistry, Faculty of Dentistry, The University of Hong Kong, Pok Fu Lam, Hong Kong; 2 Endodontology, Faculty of Dentistry, The University of Hong Kong, Pok Fu Lam, Hong Kong; 3 Paediatric Dentistry, Faculty of Dentistry, University of Otago, Dunedin, New Zealand; 4 Dental Public Health, Faculty of Dentistry, The University of Hong Kong, Pok Fu Lam, Hong Kong

**Keywords:** Biotechnology, Microbiology

## Abstract

We examined the effect of L-arginine - (i) on the growth of *L. rhamnosus* GG (LrG) and (ii) combined LrG synbiotic on the growth of cariogenic *S. mutans*. Viability of LrG was assessed using MTT/XTT assays, confocal imaging with ADS activity measurement. The effect of L-arginine (0.5%/1%/2%) (2×/24 h) with LrG on *S. mutans* was evaluated by measuring the colony forming units, biofilm biomass, real-time qPCR and confocal imaging. The pH of the spent media was measured immediately and 24 h post-treatment with assessment of lactic acid. The LrG viability was highest with 2% L-arginine (p < 0.001). Confocal imaging showed that 2% L-arginine increased biofilm thickness of LrG. The 2% L-arginine and LrG synbiotic significantly inhibited the growth of *S. mutans* (p < 0.001) reducing the viable counts (p = 0.002) and biofilm biomass (p < 0.001). The pH of spent media was the highest when treated with 2% L-arginine and LrG synbiotic (p < 0.001) with no difference between post-treatment and 24 h post-treatment (p > 0.05). Conversely, the 2% L-arginine and LrG synbiotic showed the lowest lactic acid production (p < 0.001). This study demonstrated that L-arginine enhanced the growth of LrG. The 2% L-arginine and LrG synbiotic synergistically inhibits the growth of *S. mutans* with significant potential to develop as an anti-caries regimen.

## Introduction

Dental caries, a dysbiosis-regulated biofilm-mediated disease, is an important public health problem worldwide^1^. Despite being the gold standard for caries control, fluoride has limited action on oral biofilms^2^. Recently developed strategies to combat caries focus on ecological approaches, interfering with the physical and metabolic activities of the biofilm with an objective of preventing dysbiosis. Such interventions use either antimicrobial strategies or approaches to enhance the growth of health-promoting bacteria^3^. The latter approach can be holistically summarized as a biotic means of caries prevention, which includes the use of prebiotics and probiotics.

Prebiotics, probiotics and synbiotics are well known for their beneficial health effects on the human gastrointestinal tract^4^. A prebiotic is a substrate that is selectively utilized by host microorganisms, thereby conferring a health benefit. It stimulates the growth of beneficial bacteria and suppress the growth of pathogens. Arginine has been used as an oral prebiotic to enhance the growth of alkalogenic health-promoting bacteria - *Streptococcus sanguinis, Streptococcus parasanguinis* and *Streptococcus gordonii*, with subsequent inhibition of the cariogenic bacterium - *Streptococcus mutans*^5^. Furthermore, it enhances the antimicrobial and remineralization effects of fluoride^6,7^. However, prolonged use of arginine may facilitate plaque alkalization, promoting the overgrowth of oral anaerobes such as *Porphyromonas gingivalis*^5^.

Probiotics are live microorganisms that confer a health benefit on the host when administered in adequate amounts. It provides a local protective effect by counteracting the activities of the pathogens and a systemic indirect effect on immunological amelioration. *Lactobacillus rhamnosus* GG is the world’s most researched probiotics with profound health benefits systemically and antagonistic effects on cariogenic bacteria; however, its effect in the oral environment is brief^8,9^. Recently, it has been shown that prebiotics and probiotics can potentially be developed into novel synbiotics against oral pathogens^10^ for future dental caries management^11^.

Based on such a premise, the use of synbiotics might represent a novel ecological-based caries- preventive strategy to promote the growth of healthy arginolytic bacteria *(S. sanguinis* and *S. gordonii)* by prebiotics and simultaneously enrich the growth of probiotics *L. rhamnosus* GG for enduring oral colonization. Accordingly, the objectives of the study were to examine the effect of (i) arginine on the growth of *L. rhamnosus* GG and (ii) the combined L-arginine and *L. rhamnosus* GG synbiotic on the growth of cariogenic *S. mutans*. The null hypotheses tested in the present study were: (i) L-arginine has no effect on the growth of *L. rhamnosus* GG and (ii) the combination of L-arginine with *L. rhamnosus* GG does not affect the growth of *S. mutans*.

## Results

### Effect of L-arginine on growth of probiotic L. rhamnosus GG

**Figure 1:**
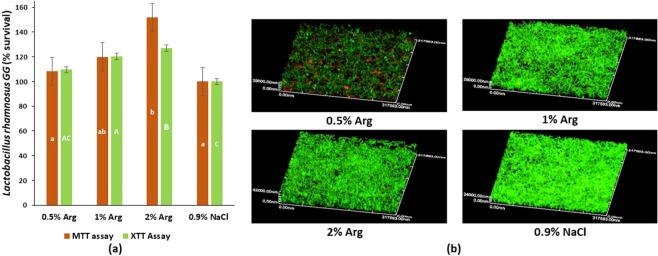
The effect of L-arginine on *L. rhamnosus* GG: (**a**) *L.rhamnosus GG* cell viability using MTT and XTT assays (Data was analyzed using Kruskal-Wallis test with Dunn-Bonferroni’s multiple comparison test at p < 0.05. The capital alphabets – A/B/C show significant difference among the groups with XTT assay and the small alphabets – a/b show significant differences among the groups with MTT assay); and (**b**) Representative confocal microscopic images (100×) of biofilms treated with 0.5%, 1%, 2% L-arginine and 0.9% NaCl.

The biofilm viability results are shown in Fig. 1a. Both MTT and XTT assays showed that the percentage survival of *L. rhamnosus* GG increased with arginine concentration. The cell viability of the *L. rhamnosus* GG with 2% L-arginine group was significantly higher than 0.5% L-arginine and the control groups (p < 0.05). Representative live/dead images are displayed in Fig. 1b. Live bacteria were stained green and dead bacteria/damaged bacterial cells were stained red with propidium iodide. Distinguishable dead/damaged bacterial cells (red staining) of *L. rhamnosus* GG could be seen in biofilms treated with 0.5% L-arginine, which were not evident in the other groups (Fig. 1b). The 2% L-arginine increased biofilm thickness of *L. rhamnosus* GG (Fig. 1b). The Arginine Deiminase System (ADS) activity measured by ammonia detection could not be determined for all the groups, suggesting that *L. rhamnosus* GG might not be metabolizing arginine through the ADS operon. The results of the first experiment demonstrated that 2% L-arginine significantly enhanced the growth of *L. rhamnosus* GG biofilm.

### Effect of L-arginine with L. rhamnosus GG on growth of cariogenic S. mutans

**Figure 2:**
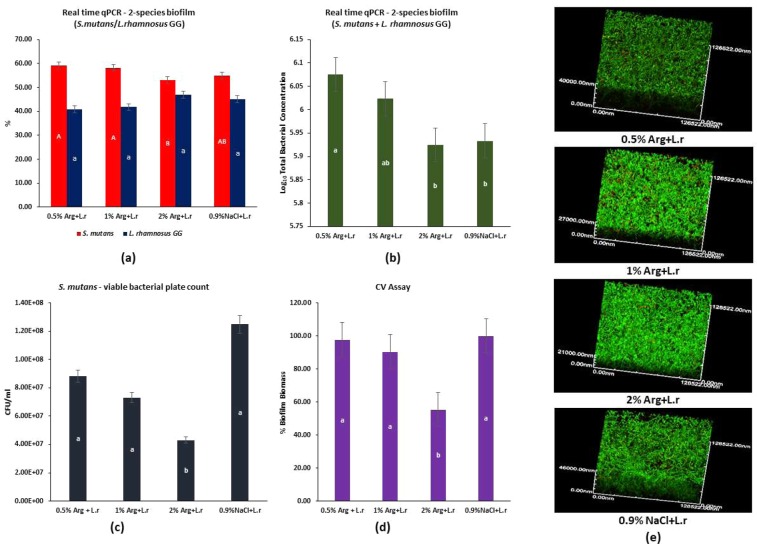
The effect of combined L-arginine and *L. rhamnosus* GG on *S. mutans*: (**a**) Proportional bacterial quantitative data of *S. mutans* (Capital alphabets – A/B indicate significant differences among the treatment groups) and *L. rhamnosus GG* (small alphabets – a/b indicate significant differences among the treatment groups) for *S. mutans* biofilms treated with L-arginine (0.5%, 1%, 2%), 0.9% NaCl and *L.rhamnosus GG*; (**b**) Total bacterial concentration estimated by real-time PCR for *S. mutans* biofilms treated with L-arginine (0.5%, 1%, 2%), 0.9% NaCl and *L.rhamnosus GG* (small alphabets – a/b indicate significant differences among the groups); (**c**) Colony forming units for *S. mutans* on agar media (small alphabets – a/b indicate significant differences among the groups); (**d**) Relative percentage of biofilm biomass measured by CV assay (small alphabets – a/b indicate significant differences among the groups); and (**e**) Representative confocal microscopic images (100×) of *S. mutans* biofilms treated with 0.5%, 1%, 2% L-arginine and 0.9% NaCl and *L rhamnosus* GG. Data for real-time qPCR and CFU were analyzed by 1-way ANOVA SNK test at p < 0.05; and data for CV assay was analyzed using Kruskal-Wallis test with Dunn-Bonferroni’s multiple comparison test at p < 0.05.

The real-time PCR results showed that the inhibitory effect of L-arginine + *L. rhamnosus* GG on *S. mutans* proportions increased with arginine concentration, with 2% L-arginine + *L. rhamnosus* GG group more evident than 0.5% L-arginine + *L. rhamnosus* GG and 1% L-arginine + *L. rhamnosus* GG groups (p < 0.001) (Fig. 2a). The total bacterial concentration for *L. rhamnosus* GG + *S. mutans* was further computed with respect to treatments with L-arginine at concentrations – 0.5%, 1%, and 2% and the control – 0.9% NaCl (Fig. 2b). The total bacterial concentration decreased with increasing arginine concentration, with the lowest observed in the 2% L-arginine + *L. rhamnosus* GG group.

Both the CFU count for *S. mutans* (Fig. 2c) and the quantified percentage biofilm biomass of *S. mutans* (Fig. 2d) decreased with increasing arginine concentration with the 2% L-arginine + *L. rhamnosus* GG group showing significantly lower biofilm biomass than the other groups (p < 0.001). Representative confocal microscopic image of biofilm revealed that the 2% L-arginine + *L. rhamnosus* GG group had noticeably reduced biofilm thickness (Fig. 2e). These results showed that the 2% L-arginine + *L. rhamnosus* GG significantly inhibited the growth of cariogenic *S. mutans*, demonstrating a synergistic synbiotic effect.

### pH measurement and generated lactic acid

**Figure 3:**
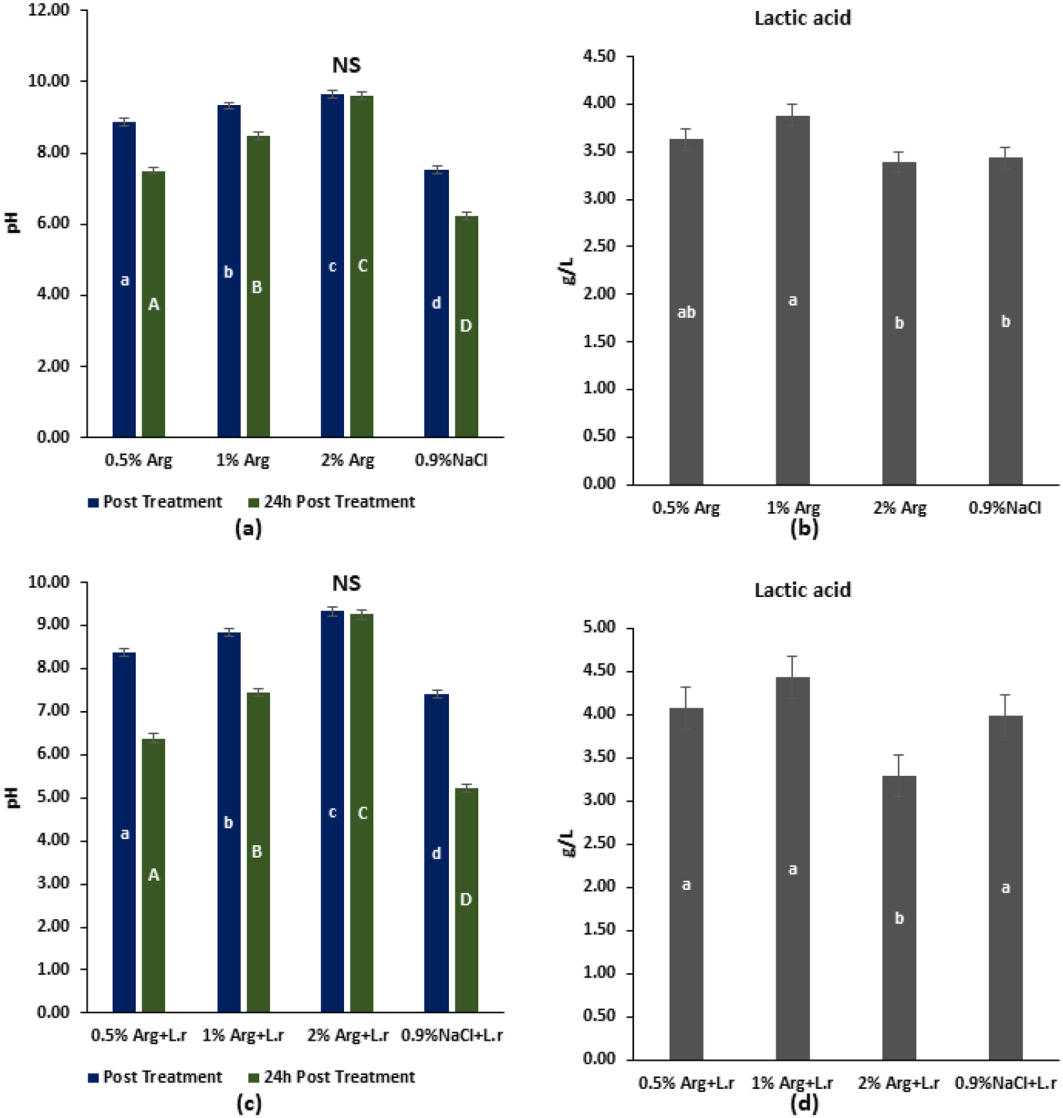
pH of spent media and lactic acid production: (**a**) Post-treatment and 24 h post-treatment pH of spent media for *L. rhamnosus* GG biofilms treated with 0.5%, 1%, 2% L-arginine and 0.9% NaCl (small alphabets – a/b/c/d show significant difference among the groups at post-treatment phase; and capital alphabets – A/B/C/D show significant difference among the groups at 24 h post-treatment phase); (**b**) Lactic acid generated in spent media for *L. rhamnosus* GG biofilms treated with 0.5%, 1%, 2% L-arginine and 0.9% NaCl (small alphabets – a/b show significant difference among the groups); (**c**) Post-treatment and 24 h post-treatment pH of spent media for *S. mutans* biofilms treated with L-arginine (0.5%, 1%, 2%), 0.9% NaCl and *L.rhamnosus GG* (small alphabets – a/b/c/d show significant difference among the groups at post-treatment phase; and capital alphabets – A/B/C/D show significant difference among the groups at 24 h post-treatment phase); and (**d**) Lactic acid generated in spent media for *S. mutans* biofilms treated with L-arginine (0.5%, 1%, 2%), 0.9% NaCl and *L. rhamnosus GG* (small alphabets – a/b show significant difference among the groups). All data were analyzed using 1-way ANOVA with SNK test at p < 0.05.

Post-treatment and 24 h post-treatment pH of the spent media of *L. rhamnosus GG* biofilms treated with L-arginine or 0.9% NaCl are shown in Fig. 3a. For both time points, pH of the spent media for *L. rhamnosus GG* biofilm increased with increasing concentration of arginine, with the highest pH observed in the 2% arginine group (p < 0.05). Lactic acid generated in the spent media for *L. rhamnosus GG* biofilms treated with different concentrations of L-arginine or 0.9% NaCl is shown in Fig. 3b. Lactic acid production was significantly lower in 2% L-arginine, 0.5% L-arginine and 0.9% NaCl groups.

Conversely, the post-treatment and 24 h post-treatment pH of the spent media of *S. mutans* biofilm treated with L-arginine and *L. rhamnosus GG or* 0.9% NaCl are shown in Fig. 3c. For both time points, pH of the spent media with *S. mutans* biofilm also increased with increasing concentration with arginine, with the highest pH in the 2% L-arginine + *L. rhamnosus* GG group (p < 0.05). Similarly, the lactic acid generated in the spent media for *S. mutans* biofilms treated with L-arginine and *L. rhamnosus GG or* 0.9% NaCl are shown in Fig. 3d. Lactic acid production was the least in 2% L-arginine + *L. rhamnosus* GG group (p < 0.05). Overall, 2% L-arginine and *L. rhamnosus GG* significantly antagonized *S. mutans* growth by creating a synbiotic synergism against *S. mutans*.

## Discussion

The present study demonstrated the feasibility and beneficial effects of a novel synbiotic with synergistic inhibitory effect on cariogenic bacterium *S. mutans*. Our results demonstrated that the use of L-arginine as a prebiotic enhanced the growth of the probiotic – *L. rhamnosus* GG in a dose-dependent manner. Thus, the null hypotheses that L-arginine has no effect on the growth of *L. rhamnosus* GG and the combined L-arginine and *L. rhamnosus* GG synbiotic does not affect the growth of *S. mutans* are to be rejected.

Probiotics are beneficial microorganisms that modulate the bacterial balance in the digestive system. For probiotics to be useful in the oral cavity, it must be able to adhere, colonize oral tissue and compete with pathogenic microorganisms at the binding site^10^. The probiotic *L. rhamnosus* GG, was used in the current study due to its ability to inhibit a large variety of human pathogenic oral bacteria, including *S. mutans*, *S. sobrinus* and *P. gingivalis*^12–14^. However, *L. rhamnosus* GG are not well-adapted for long-term persistence in the oral cavity. This problem may overcome by combining *L. rhamnosus* GG with L arginine, forming a synbiotic to improve the survival and adherence of *L. rhamnosus* GG. Recently, a study had shown that *L. rhamnosus* LRB a probiotic strain isolated from the oral cavity, similar to *L. rhamnosus* GG demonstrated a strong antimicrobial activity against pathogenic *S. mutans* and several oral streptococci^15^. Future studies can be directed to evaluate the synbiotics potential of arginine and *L. rhamnosus* LRB, as the strain is closely genetically related to *L. rhamnosus* GG with an intra-oral origin.

The preferred medium for culture of *L. rhamnosus* GG is MRS broth; while they only survive in BHI broth for short period of time. However, in this study, the growth of *L. rhamnosus* GG in BHI broth was significantly enhanced by the presence of L-arginine, with the highest percentage of survival of *L. rhamnosus* GG observed with 2% L-arginine. Due to the presence of the prebiotic arginine, *L. rhamnosus* GG acquire higher tolerance to the environmental conditions.

The effect of L-arginine and *L. rhamnosus* GG synbiotic on *S. mutans* biofilms was further assessed by evaluating the colony-forming unit counts of biofilm and biofilm biomass. The *S. mutans* viable counts and proportional biofilm biomass were significantly reduced in the 2% L-arginine + *L. rhamnosus* GG group. The results were mainly attributed to the diminished virulence of *S. mutans* as a result of the synergistic inhibitory effect of the synbiotic on both the growth and viable counts of *S. mutans*^14,16,17^.

L-arginine is known to destabilize human oral biofilms, affecting the adhesion properties of *S. mutans*^18,19^. Conversely, a number of clinical trials have shown significantly lower levels of *S. mutans* and caries incidence after consumption of the probiotic *L. rhamnosus* GG^20–22^. The inhibitory capacity of *L. rhamnosus* GG is still unclear, but has been associated with coaggregation of *S. mutans*^23^ preventing their adherence to the tooth surfaces, bactericidal effects on *S. mutans*^23^, reduced production of insoluble extracellular polysaccharides in biofilm formation^24^ and reduction of salivary counts of *S. mutans*^25^.

The real-time PCR results identified that the combined 2% L-arginine + *L. rhamnosus GG* synbiotic significantly inhibited the growth of *S. mutans*; while no significant difference in the proportion of *S. mutans* and total bacterial concentration were observed between the 2% L-arginine + *L. rhamnosus GG* synbiotic and the control groups. The reasons could be of two-folds: either the qPCR had amplified DNA from both the live and dead bacteria or the growth of bacterial cells embedded deep within the biofilm matrix had been inhibited due to matrix pressure without affecting its virulence in the control group^26^.

The pH of the spent media for *L. rhamnosus* GG was the highest and remained unchanged immediate and 24 h post treatment with 2% L-arginine. The alkaline pH environment provided by 2% L-arginine could have modulated the metabolic activities of *L. rhamnosus* GG and augmented its growth^27^. Further, *L. rhamnosus* GG has limited potential to synthesize certain amino acids (including arginine) for its growth and thus, requires exogenous supplementation of the amino acids for substantial growth and metabolic activity^28,29^. Thus, the enhanced growth of *L. rhamnosus* GG in the presence of L-arginine (at 2% concentration) is due to the active utilization of amino acid biosynthesis pathway of *L. rhamnosus* GG which thereby improves bacterial metabolism and growth^30^.

The ADS activity remained undetermined for all the groups, suggesting that *L. rhamnosus* GG could not be recognized as an arginolytic bacteria as opposed to a recently discovered novel *Streptococcus* strain A12, which demonstrated ADS activity at high levels, moderating plaque pH as well as interfering with the growth and virulence of caries pathogens, *S. mutans*. The combination of an arginine with ADS-positive probiotics into synbiotics may constitute a nonpharmaceutical advancement in caries prevention^11,31^. The pH of the spent media for *S. mutans* was the highest for 2% L-arginine *and L. rhamnosus* GG group and remained constant over time, suggesting a shift in dental plaque ecology by the synbiotic. The change in microbial ecology is also supported by lactic acid measurements, which was significantly reduced in the 2% L-arginine + *L.rhamnosus* GG group, indicating that the virulence of *S. mutans* had been affected with a general reduction in the acidogenicity of the oral microbiota. Lactic acid production by bacteria might lead to extracellular acidic pH shift, but L-arginine, either due to its inherent potential or the ammonia production through arginolytic oral commensals buffer the acidic pH. Conversely, Lin *et al*.^32^ examined the effect of probiotics on the pH of a dental biofilm following a sugar rinse among high caries-risk children aged between 7-11 years reported an increase in the pH of the biofilm and a reduction in the recovery time of pH with a short-term probiotics intake.

**Figure 4:**
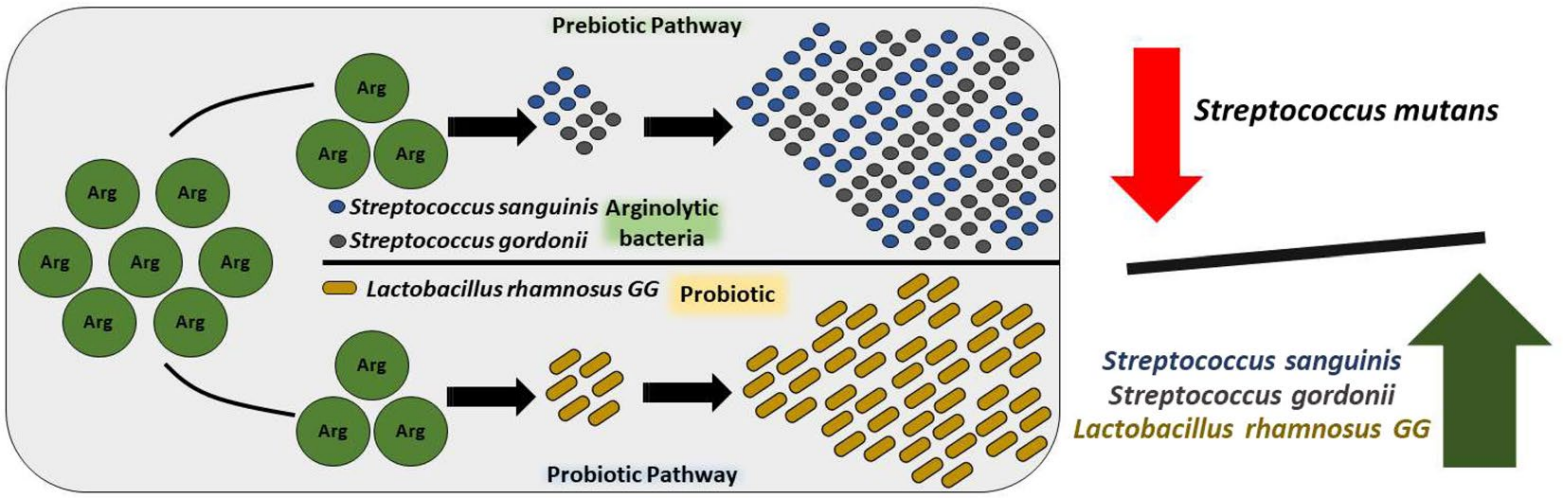
*Ab initio* contemplated mechanism of action.

The *ab initio* contemplated model on the mechanism of actions of the synbiotics and the beneficial effects associated with the synbiotic administration are presented in Fig. 4. We propose that the L-arginine molecules deliver its ecological-based caries-preventive treatment through two pathways. The first pathway is the prebiotic effect on the oral arginolytic commensals – *S. sanguinis* and *S. gordonii*, whereby arginine augments the growth of the arginolytic bacteria and affect the oral biofilm ecology^33–35^. In the prebiotic pathway, L-arginine will be metabolized by arginolytic commensals into ammonia and ATP through the ADS operon^36,37^. The health-associated commensals utilize the ATP for their survival, help in maintaining homeostasis and creating a non-conducive environment for cariogenic *S. mutans*^38^. Additionally, the probiotic *L.rhamnosus* GG utilizes L-arginine, creating a stable environment conducive for its growth. Thus, the probiotic pathway supplements the brief availability of *L. rhamnosus* GG in the oral cavity, thereby augmenting its sustenance^8,9^.

The increased sustainability of the probiotic bacteriotherapy will enhance the pathogenic colonization resistance and immune modulation potential^16^. The synbiotic therapy through its synergistic mechanism will further assist in reducing the pathogenic biofilm formation^39^. Thus, the synbiotic therapy of L-arginine combined *L. rhamnosus* GG potentially enhances a metabolic interactive organization in oral biofilms, that could physiologically generate microbial colonies with increased homeostasis^40^. Overall, the synbiotic therapy will serve as an oral biofilm modifier and can be an effective anti-caries treatment, especially for high caries-risk patients that addresses the much-needed ecological-based caries preventive approach^3^. Such synbiotics can be incorporated into mouth rinses or dentifrice for daily use. The twice daily use of the synbiotics might help maintain ecological homeostasis in patients after meal consumption, whereby the low plaque pH and its detrimental cariogenic effects can be prevented. Apart from anti-caries benefits, this combination might have some potential systemic benefits, as both L-arginine and the prebiotic *L. rhamnosus* GG have demonstrated systemic benefits individually.

This study showed that synbiotics, having both prebiotics and probiotics properties, are more effective than probiotics alone in antagonizing the cariogenic bacterium *S. mutans*. However, the results should be viewed with caution, as the study uses a mono-species biofilm in a closed microbial (microtiter biofilm) model as compared to the open model that undertakes a continuous culture system with pulsed treatment. Future studies should investigate the effects of synbiotics, alone and in combination with fluorides, on polymicrobial models, to provide conclusive recommendations for clinical use.

## Methods

Culturelle (i-Health, Inc., Denmark) – a probiotic dietary supplement was routinely cultured at 37 °C for 72 h under anaerobic conditions (85% N_2_, 10% H_2_, 5% CO_2_) to obtain *L. rhamnosus* GG in BHI broth (pH-7). The cells were then adjusted to a concentration of 10^7^ cells/ml in BHI broth (pH-7) with concentration estimated using UV-Vis Spectrophotometer (Beckman Coulter, CA, USA) at 660 nm. The strain was matched to an in-house *L. rhamnosus* GG ATCC 53103 using real-time quantitative polymerase chain reaction (qPCR) and was found to conform to the reference strain. *S. mutans* UA159 (ATCC 700610) was similarly cultured as *L. rhamnosus* GG and adjusted to a concentration of 10^7^ cells/ml in BHI broth. The L-arginine treatments were disposed at concentrations −0.5 *w/v* %, 1 *w/v* %, and 2 *w/v* % in 0.9% NaCl; whereby 0.9% NaCl was treated as control. For all experiments, prior to biofilm analysis, the wells were washed with PBS twice.

For the first experiment, *L. rhamnosus* GG cells were cultured in a 96-well microplate to receive the 1^st^ L-arginine treatment. The second treatment of test solutions were added after 4 h during the 24 h incubation period in an anaerobic chamber (85% N_2_, 10% H_2_, 5% CO_2_). After 24 h, the spent media was removed to analyze biofilm using MTT and XTT assays for probiotic cell viability, ADS activity, and confocal laser scanning microscopy (CLSM).

For the second experiment, *S. mutans* cells were primarily cultured in a 96-well microplate. The treatment in this section, comprised of *L. rhamnosus* GG and pre-determined concentrations of L-arginine combined at 2-different segments 4 h apart each. The treated wells were incubated in an anaerobic chamber (85% N_2_, 10% H_2_, 5% CO_2_) for 24 h. After the incubation period, the biofilms were analyzed by counting colony forming units for *S. mutans* on spiral plated blood agar plates, biofilm biomass determination using crystal violet (CV) assay, and real-time qPCR to determine proportional bacterial cells for *S. mutans* (*a priori* to test the hypothesis) and *L. rhamnosus* GG with total bacterial concentration in the biofilm.

For both sets of experiments, pH of the spent media was measured and the amount of lactic acid generated in the media was quantified.

### Probiotic cell viability

The probiotic *L. rhamnosus* GG cell viability was assessed using MTT and XTT assays after 24 h incubation as per a previous study^41^. For the probiotic cell viability assay, the spent media was removed followed by biofilm wash with PBS.

MTT reagent was dissolved in PBS and dispensed on *L. rhamnosus* GG biofilm. The reagent with biofilm was incubated for 4 h at 37 °C. After 4 h, MTT solvent – dimethyl sulfoxide (DMSO) was added and incubated for 20 min on an orbital shaker at room temperature. The viability was analyzed with a microplate reader at 590 nm.

The reagent was prepared by combining XTT Developer Reagent and Electron Mediator Solution with a prior thawing of all components at room temperature. The prepared agent was further introduced to the biofilms and mixed for 5 min on an orbital shaker at room temperature. Further, the biofilms treated with XTT reagent were incubated at 37 °C in a CO_2_ incubator for 4 h. Prior to reading the plate, the reduced XTT dye was allowed to mix on an orbital shaker for 5 min for homogenization. Following which the mix was removed in an Eppendorf tube to centrifuge at 10,000 rpm for 15 min. The supernatant was dispensed in a microplate to measure absorbance at 450 nm^41^. All obtained OD values for MTT and XTT assay were normalized to the respective controls for further analysis.

### Confocal laser scanning microscopy

Qualitative assessment of *L. rhamnosus* GG biofilm grown with twice-daily treated L-arginine was evaluated using CLSM. For the purpose of this experiment, the biofilms were grown on hydroxyapatite (HA) discs (5 mm × 2 mm; Clarkson Chromatography Products, Inc., USA) instead of microplate which were dip-washed in PBS prior to staining with LIVE/DEAD BacLight (SYTO 9/propidium iodide) Kit (Invitrogen detection technologies, USA) for 30 min at room temperature. The biofilms were scanned with two-photon laser scanning microscope (Olympus, FLUOVIEW FV1000, USA) at excitation/emission wavelengths for SYTO 9 – 480/500 nm and Propidium Iodide – 490/635 nm. The biofilms were scanned at 3 randomly selected view fields at 100-x magnification. Similarly, to examine the effect of combined L-arginine (at concentrations – 0.5%, 1%, and 2%, respectively) with *L. rhamnosus* GG on *S. mutans* biofilm, the bacterial cells were inoculated on HA discs and the biofilms were eventually stained to scan random 3-view fields at 100-x with CLSM.

### Colony forming units

The total number of *S. mutans* viable cells in the second experiment was determined by counting CFU on spiral plated (Autoplate 4000, Spiral Biotech Inc., MA, USA) biofilm suspension in BHI broth on Horse Blood Agar (HBA). The HBA plates with biofilm suspensions were incubated for 72 h anaerobically prior to CFU counting. The CFU counting for each suspension was determined based on the diluted suspensions approximated and grown on the agar plates identified in the feasibility study phases of the experimental protocol.

### Biofilm biomass

Biofilm biomass was determined by crystal violet (CV) staining assay as per previous studies^42,43^. The PBS washed biofilms from the second experiment were fixed with methanol for 5 min which was eventually allowed to dry for 15 min. The fixed biofilms were then stained with 0.1% CV stain for 15 min. After staining, the biofilms were washed with PBS thrice and dried for 2 h until complete evaporation of moisture. The dye was then extracted with 95% ethanol and absolute acetone for 5 min. The solution was allowed to homogenize prior to reading with microplate for absorbance at 570 nm. The data was normalized to the control.

### Arginine deiminase system (ADS) activity

ADS activity (as µmol of ammonia) was measured by quantification of ammonia generated from the incubation of biofilm with Nessler’s reagent at 37 °C as per a previous study^44^. The ammonia produced was detected by Nessler’s reagent (Sigma-Aldrich, St. Louis, USA) using 8-point reference of ammonium sulfate (10 mM high-point reference) subjected to a standard curve (R^2^ = 0.999). Each sample was assayed in triplicate. ADS activity determined was recorded as µmol of ammonia liberated in the biofilm.

### pH measurement

The pH of spent media (BHI) was determined using pH electrode (CyberScan pH 500, Thermo Scientific, USA) calibrated to standard solutions (4.01, 7.00, 10.01). For the purpose of pH evaluation, respective inoculums were introduced in a sterile container to receive the electrode for assessment. The pH was determined at two time-points – post intervention treatment and after 24 h incubation in the anaerobic chamber (85% N_2_, 10% H_2_, 5% CO_2_). Prior to each measurement, the electrode was first rinsed with DI water and then thoroughly disinfected with 70% *v/v*. ethanol. The instrument was re-calibrated at intervals preferably between group measurements to achieve a stand-alone optimum electrode standardization for pH measurements.

### Lactic acid determination

Lactic acid (in g/L) generated post 24 h incubation in anaerobic chamber was determined using a spectrophotometric method as per a previous study^45^. The spent media was centrifuged at 10,000 g for 5 min. The supernatant was drawn as a test solution to determine lactic acid. Briefly, the supernatant was added to 0.2% FeCl_3_ solution and vortexed for 60 s. The absorbance of the working solution was measured at 390 nm against a series of reference solutions derived standard curve (R^2^ = 0.999). The lactic acid concentration was calculated based on the standard curve to obtain measurements in g/L.

### Quantitative polymerase chain reaction (qPCR)

The molecular based qPCR determination of total bacterial cells was primarily used to examine the *S. mutans* and then proportional *S. mutans* and *L. rhamnosus* GG cells in the second experiment that highlights the effect of the proposed therapy on cariogenic *S. mutans*, considering it as a dual-species biofilm. The DNA quantification (in CFU/ml) was done as per our previous study^7^.

Biofilms were transferred to PBS in an Eppendorf tube which were further centrifuged at 14,000 rpm for 10 min. Following which the supernatant was discarded and the bacterial pellet was suspended in 20 mM Tris-HCl, pH: 8.0; 2 mM EDTA; 1.2% Triton and cells lysed with 20 mg/ml lysozyme incubated in a water bath at 37 °C overnight. The genomic isolation of DNA was performed using QIAamp DNA Isolation kit (Qiagen, Hilden, Germany) following manufacturer instructions. *S. mutans* ATCC 700610 and *L. rhamnosus* GG ATCC 53103 isolated DNA’s served as positive controls for the reaction.

The oligonucleotide primers used in the present study for *S. mutans* and *L. rhamnosus* GG were F: 5′-GCCTACAGCTCAGAGATGCTATTCT-3′; R: 5′-GCCATACACCACTCATGAATTGA-3′ and F: 5′-CAGAAATCAAAGAAGACAAACTCGTTA-3′; R: 5′-CCATGTAAACGGACAATGGGAGT-3′^14^; respectively. Similarly, the Taqman probes (Applied Biosystems, USA) for *S. mutans* and *L. rhamnosus* GG used in the present study were 5′-FAM-TGGAAATGACGGTCGCCGTTATGAA-TAMRA-3′ and 5′-6FAM-CGGATTTCCAAAGCAATTCTTAACGATGAAAATG-TAMRA-3′^14^; respectively. The probes and primers were mixed with Taqman universal PCR mix and isolated DNA for the reaction.

The cycle condition for the reaction was set as follows: 50 °C/2 mins; 95 °C/10 mins; 50 cycles of 95 °C/15 sec and 58 °C/1 min. The standard curve was obtained from the known concentrations of isolated DNA from the respective positive controls whereas the negative control was sterilized Milli-Q water instead of isolated DNA from the samples/controls. The reaction was performed using Step One Plus (Applied Biosystems, USA) subjected to description of appropriate controls and reaction initiated in a microAMP optical PCR reaction plate for the system.

### Statistical analysis

All experiments were performed at least in triplicate. The statistical analysis for the parametric data was performed using 1-way ANOVA followed by SNK test; whereas for non-parametric data the analysis was done using Kruskal-Wallis test with Dunn-Bonferroni’s multiple comparison test. Post-treatment and 24 h post-treatment pH measurements were compared with paired sample t-test. For both data categories, the statistical significance was set at p < 0.05.

## Conclusion

This study highlights that L-arginine enhances the growth of *Lactobacillus rhamnosus GG*. The combined 2% L-arginine and *Lactobacillus rhamnosus GG* synbiotic synergistically inhibits the growth of *Streptococcus mutans* and has significant potential to develop as an anti-caries regimen.
